# Sweaters and Other Comforts

**Published:** 1917-09-29

**Authors:** John Penoyre

**Affiliations:** 8 King's Bench Walk, Inner Temple, E.C. 4.


### Sweaters and Other Comforts.

*To the Editor of* THE HOSPITAL.

SIR,—Are your good readers going to prove good friends to "sweaters" again for this the fourth winter of the war? We have made a good start with 10,999 comforts sent me as the result of a summer knitting appeal.

The sweaters and other things have outgrown this garret, so will your readers kindly address the sacks and parcels to me, c/o Sir Edward Ward, D.G.V.O., 45 Horseferry Road, S.W. 1? They will have heard all about Sir Edward's Central Pool of Comforts with its bases on every Front, from which officers can draw direct for exactly what their men need. Waste and delay are thus avoided, but it is a point of honour for us all to keep these base depôts well supplied throughout the winter. I know all about the wool difficulty, but we can but do our best and be of good cheer, bearing in mind that this may be the last winter of the war.

I have here at 8 King's Bench Walk, Temple, E.C. 4, plenty of easily knitted printed patterns of all comforts, for any ladies who like to write for them. They are, of course, free, but it is considerate to enclose stamps to cover postage.—Yours faithfully, JOHN PENOYRE.

8 King's Bench Walk, Inner Temple, E.C. 4.
September 20, 1917.

